# Gut-Microbiome Composition in Response to Phenylketonuria Depends on Dietary Phenylalanine in BTBR Pah^enu2^ Mice

**DOI:** 10.3389/fnut.2021.735366

**Published:** 2022-01-04

**Authors:** Els van der Goot, Stefanie N. Vink, Danique van Vliet, Francjan J. van Spronsen, Joana Falcao Salles, Eddy A. van der Zee

**Affiliations:** ^1^Molecular Neurobiology, Groningen Institute for Evolutionary Sciences, University of Groningen, Groningen, Netherlands; ^2^Microbial Ecology Cluster, Groningen Institute for Evolutionary Sciences, University of Groningen, Groningen, Netherlands; ^3^Department of Pediatrics, Beatrix Children's Hospital, University Medical Center Groningen, Groningen, Netherlands

**Keywords:** phenylketonuria (PKU), gut-microbiome, behavior and cognition, phenylalanine restriction, diet

## Abstract

Phenylketonuria (PKU) is a metabolic disorder caused by a hepatic enzyme deficiency causing high blood and brain levels of the amino acid Phenylalanine (Phe), leading to severe cognitive and psychological deficits that can be prevented, but not completely, by dietary treatment. The behavioral outcome of PKU could be affected by the gut-microbiome-brain axis, as diet is one of the major drivers of the gut microbiome composition. Gut-microbiome alterations have been reported in treated patients with PKU, although the question remains whether this is due to PKU, the dietary treatment, or their interaction. We, therefore, examined the effects of dietary Phe restriction on gut-microbiome composition and relationships with behavioral outcome in mice. Male and female BTBR Pah^enu2^ mice received either a control diet (normal protein, “high” Phe), liberalized Phe-restricted (33% natural protein restriction), or severe Phe-restricted (75% natural protein restriction) diet with protein substitutes for 10 weeks (*n* = 14 per group). Their behavioral performance was examined in an open field test, novel and spatial object location tests, and a balance beam. Fecal samples were collected and sequenced for the bacterial 16S ribosomal RNA (rRNA) region. Results indicated that PKU on a high Phe diet reduced Shannon diversity significantly and altered the microbiome composition compared with wild-type animals. Phe-restriction prevented this loss in Shannon diversity but changed community composition even more than the high-Phe diet, depending on the severity of the restriction. Moreover, on a taxonomic level, we observed the highest number of differentially abundant genera in animals that received 75% Phe-restriction. Based on correlation analyses with differentially abundant taxa, the families *Entereococacceae, Erysipelotrichaceae, Porphyromonadaceae*, and the genus *Alloprevotella* showed interesting relationships with either plasma Phe levels and/or object memory. According to our results, these bacterial taxa could be good candidates to start examining the microbial metabolic potential and probiotic properties in the context of PKU. We conclude that PKU leads to an altered gut microbiome composition in mice, which is least severe on a liberalized Phe-restricted diet. This may suggest that the current Phe-restricted diet for PKU patients could be optimized by taking dietary effects on the microbiome into account.

## Introduction

Phenylketonuria (PKU) is a rare inborn disease caused by faulty amino acid metabolism, with a prevalence of ~1:10,000 people, depending on geographic location (1, 2). PKU is characterized by dramatically increased levels of the essential amino acid phenylalanine (Phe) due to a deficiency in the hepatic enzyme Phe hydroxylase. Clinically, untreated PKU leads to severe intellectual disability, anxiety and depression disorders, motoric problems, epilepsy, and seizures. Since the 1960s, the most severe symptoms have been prevented by new-born screening programs and the implementation of a low-Phe diet. When started early and adhered to continuously, this treatment is very effective in keeping Phe levels within an acceptable range (120–360 μmol/L for children) by restricting the intake of natural protein-rich in Phe, while supplementing with amino acids and essential micronutrients to avoid deficiencies (1, 3). Nevertheless, it has been shown that despite treatment, variations in the neurocognitive, psychosocial, and metabolic outcome of PKU remain (4–7), while maintaining Phe levels of 120–360 μmol/L, or even 120–600 μmol/L, is very difficult for adolescents and adults (1).

The restricted dietary treatment of PKU also has consequences for the composition of the microbes present in the digestive tract, i.e., the gut microbiome. In general, the gut microbiome is a highly variable and unique community that interacts with the host's physiology, thus influencing host phenotype and fitness by producing many metabolites (8, 9). For example, it is known that the gut microbiome plays a role in nutrient metabolism, priming of the immune system, and even seems to be capable of influencing host behavior (10–14). Although the microbiome is sometimes considered a “virtual organ” (9), it must be realized that it consists of individual species that are constantly competing for resources that become available either through host nutrition or as by-products generated by microbe metabolism (15–17). The biotic interactions between microbial species, together with the availability of food resources, exert selective pressures, playing an important role in determining the composition, and functionality of the microbiome of a given host (18–20). In PKU, both resource availability and altered host metabolism could significantly impact the microbiome composition and functionality.

One of the first thorough studies that examined microbiome composition in (young) patients with PKU was published by De Oliveira et al. (21), who compared the gut bacterial communities of eight young patients with PKU on a Phe-restricted diet with that of 10 healthy individuals using 16S ribosomal RNA (rRNA) gene sequencing. Results indicated that PKU patients showed lower bacterial diversity and, therefore, potentially reduced microbiome functionality (i.e., functions carried out by the microbes in our microbiome) related to processes associated with starch/glucose and amino acid metabolism (21). Other studies on PKU in both mice and young children also suggest a decrease in diversity and evenness of the gut microbiome when compared with the non-PKU controls (22–24). The first paper examining the adult microbiome in patients with PKU was recently published (25), revealing that the adult PKU microbiome differs from the microbiome in PKU children. However, the question remains whether the observed changes are innate to the disease, a consequence of (nutritional) treatment, or an interaction between the two [but see the study from Bassanini et al. (24) showing a difference in microbiome composition between treated patients with PKU and untreated patients with mild hyperphenylalaninemia], highlighting the need for further research.

To address this question, we conducted an experiment in which we fed PKU mutant mice diets with different natural protein contents (supplemented with amino acids) to restrict their Phe-intake and investigated the change in the microbiome composition in response to these diets. We hypothesized that, compared with the wildtype (WT) mice, PKU mutant mice have an altered microbiome composition due to their metabolic disorder. Additionally, we expect that this change in the microbiome composition could be exacerbated by limiting the availability of specific resources as a result of dietary restrictions. From an evolutionary perspective, the restriction of Phe is likely to select against bacterial species capable of utilizing and/or degrading Phe, as this resource is highly restricted, limiting chances of survival of microbes that rely on this resource. By manipulating the dietary Phe intake, we create a gradient of plasma Phe and examine how dietary Phe restriction creates selection pressure toward different microbes. Although exploratory, we predict that the microbiome of a host with PKU has adapted to the high Phe environment and that a Phe-restricted diet will therefore result in a “mismatch.” Moreover, as host-microbiome and microbe-microbe interactions within the host play an important role in the host physiology and behavior, we correlated the microbiome data to the behavioral test outcomes to verify whether variations in the behavioral outcome and metabolic control associated with patients with PKU (4–7) could potentially be explained by the microbiome composition. Increasing our knowledge on the effects of dietary Phe content on the microbiome composition could therefore provide insights about new therapeutic approaches for improved treatment of PKU.

## Materials and Methods

### Breeding and Genotyping

Original breeding pairs of Black and Tan Brachyury (BTBR) Pah^enu2^ mice were regularly backcrossed with BTBR WT mice. Heterozygous breeding pairs were used to obtain homozygous WT and homozygous Pah^enu2^ (PKU) mice. Pups were weaned at postnatal day 28, and the tissue from their ear clips was collected to establish genotype with quantitative PCR (qPCR). The tissue was incubated with a lysis buffer [100 mM Tris-HCL (Sigma-Aldrich Chemie B.V., Zwijndrecht, The Netherlands) (pH 8.5), 5 mM Ethylenediaminetetraacetic acid (EDTA) (Sigma-Aldrich Chemie B.V., Zwijndrecht, The Netherlands) (pH 8.0), 200 mM NaCl (Sigma-Aldrich Chemie B.V., Zwijndrecht, The Netherlands), 2% SDS (Bio-Rad Laboratories B.V., Lunteren, The Netherlands)], and proteinase K (Merck Chemicals B.V., Amsterdam, The Netherlands) [100:1, volume:volume (v:v)] overnight. The samples were then centrifuged (13.000 rpm × 10 min), the supernatant was transferred to clean tubes, and isopropanol (Sigma-Aldrich Chemie B.V., Zwijndrecht, The Netherlands) was added (1:1). The samples were centrifuged a second time (6.000 rpm × 10 min), after which the supernatant was discarded, and the DNA samples were dried before adding 200 μl Tris-HCl-EDTA-buffer (pH 8.0) to each sample. Genetic characterization was performed using qPCR analysis with primers aimed at exon 7 of the PAH gene (forward primer: 5′ CCGTCC TGTTGCTGGCTTAC 3′, reverse primer: 5′ CAGGTGTGTACA TGGGCTTAGATC 3′), and 5 μM probes tagged with a FAM fluorophore (WT probe: CCGAGTCZZLCALTGCA) and a Yakima Yellow fluorophore (PKU probe: CCGAGTCZLLCACTGCA) obtained from Eurogentec (Seraing, Belgium), using SensiMix™ II (GC Biotech B.V., Waddinxveen, the Netherlands) with ROX. The qPCR consisted of a 1 min cycle at 60°C, a 10 min cycle at 95°C, and 40 cycles of 15 s heating at 95°C and 1 min cooling at 60°C.

The animals were housed at 21 ± 1°C on a 12-h light-dark cycle (lights on, 08:00–20:00) with *ad libitum* access to water and food (Altromin, Lage, Germany 1414 mod.–NL_141004). Before inclusion in the experiment, the animals were group-housed per litter and randomly assigned to one of the four experimental conditions between postnatal days 28 and 35. During the experiment, the animals were individually housed with a paper roll and nesting material and *ad libitum* access to food and water. The study was approved by the Institutional Animal Care and Use Committee of the University of Groningen (DEC Number: 6926A).

### Experimental Design

In total, 56 animals were used: 14 WT (7 males and females) and 42 PKU (14 per group, 7 males, and 7 females). The animals received *ad libitum* access to either a control (the WT and PKU high Phe groups) or (semi) Phe-restricted diet (2 experimental PKU groups) for 10 weeks. Their food intake, water intake, and body weight were monitored daily during the first week and once a week after. The fecal samples for microbiome analysis were individually collected before the start of the dietary treatment (0 weeks), after 6 weeks, and during termination (10 weeks). The behavioral assessment took place during weeks 8 and 9 of the dietary treatment. After 10 weeks, the animals were terminated and plasma samples were collected for amino acid analysis.

### Experimental Diets

The basal diet used for all the interventions was AIN-93M (124 g/kg diet protein), given to the PKU high Phe and WT groups in unadjusted form. For the Phe-restricted groups, casein was reduced by 33% and 75% (Phe-restricted) and compensated by a synthetic amino acid mixture (without Phe). Due to the reduced uptake of synthetic amino acids compared with protein, an assumed protein conversion factor was taken into account, and 20% extra amino acid mixture was added at the expense of cornstarch [For details about the content of the diets, see Supplementary Table 1, which has been adapted from a study by van Vliet et al. (26)]. Diets were prepared by Research Diet Services B.V. (Wijk bij Duurstede, The Netherlands).

### Behavioral Testing

Tests were performed between 1 and 5 h after the start of the light phase. The following tests were used, in order, with three resting days between tests: the open field (OF) test to assess exploratory behavior, the novel object recognition (NOR) test, and spatial object recognition (SOR) test for learning and memory assessment (cognition), and the balance beam (BB) to test motor performance. Before each test, the animals were habituated in the experimental room for 2 min. For the OF, NOR, and SOR, the same square arena was used (50 × 50 × 35 cm) with a white Plexiglas floor, gray Plexiglas walls, and a checkerboard cue one of the walls. The trials were video recorded from above and later analyzed with Ethovision tracking software version 11 (Noldus Information Technology B.V., Wageningen, Netherlands) to track the movement and exploration of the different objects. During testing, dim lighting was used (10 lux in the center of the arena). After each trial, the animals were placed in their home cage, and the arena and objects were cleaned with 30% ethanol. For the balance beam, the trials were recorded from behind the beam and later manually analyzed for the time the animals needed to cross the beam and the number of correct steps. A more detailed description of the tests can be found in Bruinenberg et al. (27).

### Sample Collection and Sequencing Analysis

Fecal samples were collected for each mouse individually at 0, 6, and 10 weeks of treatment. At the 0 and 6 weeks of treatment, fecal samples were collected by taking fresh fecal pellets during cleaning of the cages to disturb the animals minimally. At termination (10 weeks), feces were directly collected from the lower gut. The feces were placed in Eppendorf tubes and stored at −20°C until further processing.

The fecal samples were processed for sequencing by isolating the DNA with the Qiagen, Venlo, the Netherlands PowerSoil DNA extraction kit, and DNA concentrations were adjusted to 20 ng/μl. The V4–V6 regions of the 16SrRNA gene were amplified by qPCR (forward primer: 16S-0515F-M 3′ TGYCAGCMGCCGCGGTA 3′, reverse primer: 16S-926R 5′ CCGYCAATTYMTTTRAGTTT 5′) (28) to obtain 50 ng/0.5 μg of the PCR product. The PCR product was purified using the GeneJet purification kit and adjusted to 30 ng/μL. All sequences in each sample were labeled with a unique 10 base pair barcode, after which samples were pooled and sequenced bi-directionally (2 × 300 bp) on the Illumina MiSeq platform at Genewiz (Takeley, the United Kingdom).

Raw sequencing data were demultiplexed and processed using cutadapt and QIIME2/2018.8 to remove primers and barcodes from the sequences, using the high-performance peregrine cluster of the University of Groningen (29, 30). Since the low sequence quality at the 3' end of the forward and reverse reads precluded pairing, the sequences were trimmed at base 196. The resulting forward reads were sufficient in length to analyze the highly variable V4 region but not the V5–V6 regions. The sequencing errors were identified and denoised using Deblur, resulting in amplicon sequence variants (ASV) that are inferred to have a true biological origin (i.e., originate from bacterial species) (31). Taxonomy was assigned using the RDP-Classifier v.2.12 (32) with a confidence cut-off of 0.70. A midpoint-rooted phylogenetic tree was constructed using QIIME2 v.2018.8, after which the data was imported in R version 4.0.4 (33) using qiime2R v.0.99.4 (34) and further analyzed with the Phyloseq package v.1.34.0 (35). Singletons were removed and the archaea, chloroplast, and mitochondrial sequences were filtered from the dataset. All samples were rarefied to 4,498 reads per sample using the Phyloseq package, which led to the loss of five samples divided overall groups and time points. The final set contained 2,220 taxa by seven taxonomic ranks and 148 samples.

### Statistical Analyses

#### Behavioral Outcome

The time spent in the center of the arena (%) for the OF, the time spent exploring the novel or displaced object (%) for the NOR and SOR, and the percentage of good steps and slips for the BB were calculated. Cook's distance was used to estimate and identify the influential data points in the same manner for the microbiome analyses (six times the mean of the Cook's Distance for each parameter). Normal distribution and homogeneity of variance were assessed using the Shapiro-Wilk test and Levene's test, respectively. The effect of diet and sex was then analyzed with a two-way ANOVA. A significant ANOVA was followed by *post hoc* pairwise comparisons with a pairwise *t*-test with an overall *p*-value false discovery rate (FDR) correction for all behavioral parameters. Lastly, for the NOR and SOR, the ability to master the learning task was assessed using a one-sample *t*-test against chance level (μ = 50).

#### Alpha Diversity

Alpha diversity was examined by calculating observed ASV richness, Shannon-Wiener diversity index (evenness of sequence variance distribution), and Simpson diversity index (weighted proportional abundance and richness) using the Phyloseq package (35), Pielou's evenness index [microbiome package (36, 37)] and Faith's Phylogenetic Diversity using Picante (38) and btools (39), for each experimental group. Cook's distance was used to estimate and identify the influential data points, equal to four times the mean of Cook's distance for each parameter. After evaluation, the extremes based on six times the mean of Cook's distance were removed to avoid the removal of data point close to the cut-off value. The data were tested for normal distribution using the Shapiro-Wilk test, and Levene's test was used to assess the homogeneity of variance. A two-way ANOVA for “Diet” and “Sex,” or Kruskal-Wallis in case of non-normal distributed data was conducted to compare the diets within each time point or changes over time for each diet separately. A significant ANOVA or Kruskal-Wallis test followed *post hoc* pairwise comparisons with a pairwise *t*-test or Wilcox test. An overall *p*-value correction was performed for all alpha-diversity results using the false discovery rate (FDR) correction method.

#### Beta Diversity

Principal coordinate analysis (PCoA) using Bray-Curtis dissimilarity was used to visualize the variation in the bacterial community composition between the experimental groups. Differences between bacterial communities were analyzed by Permutational Multivariate ANOVA (PERMANOVA) with diet, time point, and sex as factors and mouse as covariate (vegan package v.2.5-7) (40), followed by *post hoc* pairwise multilevel comparisons (pairwiseAdonis package) (41), to analyze the differences in the communities between diets for each time point and between time points for each diet. The Bray-Curtis multivariate dispersion (the amount of variation within each group expressed as the average Bray-Curtis distance from the median of the group) was calculated using the vegan package, and the change in beta-diversity over time depending on diet was determined by calculating the difference between time point 1 (Start) and time point 2 (6 weeks), and time point 2 and time point 3 (10 weeks), for each mouse, respectively. Outlier identification and tests for normality and homogeneity of variance were performed as described under “alpha-diversity.” The multivariate dispersion and change in beta-diversity over time data were analyzed using a two-way ANOVA for diet and sex, followed by a *post hoc* permutation test for constrained correspondence analysis (multivariate dispersion), or a pairwise *t*-test or Kruskal-Wallis test (change in beta-diversity over time), *p*-values for each variable were adjusted using the FDR correction.

#### Taxonomic Classification

To identify differences in the presence and number of ASVs, pairwise comparisons between taxonomic unit counts were made between groups at each respective time point and between time points for each diet with a differential expression analysis based on the Negative Binomial distribution using the DESeq2 package (42). Comparisons were made to examine the effect of PKU (PKU high Phe vs. WT) and treatment (Phe-Restricted group vs. either the WT or PKU High Phe) and lastly, how a semi Phe-Restricted diet relates to either treated or untreated PKU (Semi Phe-restricted vs. either Phe-restricted or PKU high Phe). Differential expression analyses were performed at the phylum, family, and genus levels.

#### Correlation Analyses

Differentially expressed families and genera were correlated with the plasma Phe levels, collected during the termination and behavioral outcome. As DESeq uses Cook's distance to identify and process influential data points, the Phe levels and count data were handled as previously described for our other variables. Correlation analyses were performed using the Spearman correlation analysis. Correlations with behavior and Phe levels were corrected separately using FDR correction, both unadjusted and adjusted *p*-values will be reported.

## Results

### General Health and Dietary Intake

The experimental diets were well-tolerated by all mice based on the food intake and growth of the mice. One PKU male on the control diet was euthanized during the experiment, and one female unexpectedly died in the 6th week. Postmortem macroscopic examination did not reveal obvious pathology.

**Figure 1:**
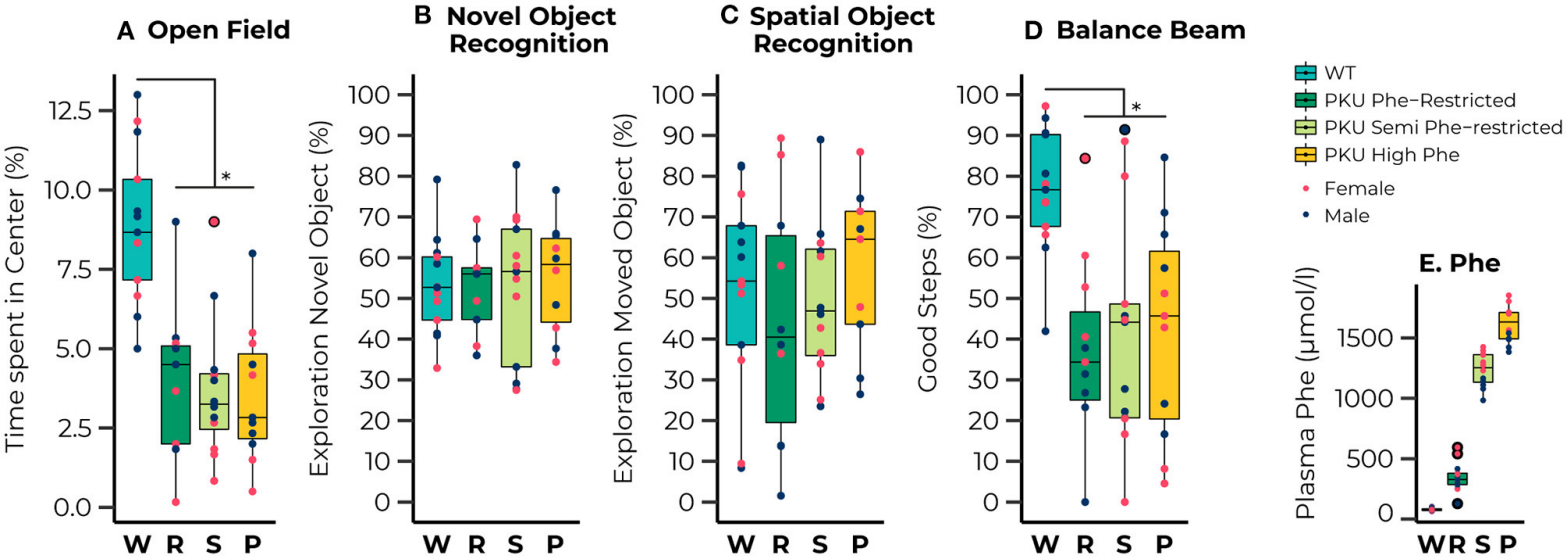
Results of the behavioral testing of wild-type (WT) animals (blue) and Phenylketonuria (PKU) animals on a Phenylalanine (Phe)-Restricted (dark green), semi-Phe-restricted (light green), or high Phe diet (yellow) including average Phe levels measured for each group (bottom right). A PKU phenotype was observed for the percentage of time spent in the center of the open field [Diet; *F*_(3,39)_ = 14.93, *p* < 0.001;all comparisons with WT, *p* < 0.001] and the percentage of good steps placed on the Balance Beam {[Diet; *F*_(3,34)_ = 7.77, *p* < 0.001; all comparisons with WT, *p* = 0.002]. No significant differences were observed in learning and memory performance; none of the groups could master the learning task. Results are displayed as the median (50th percentile) and interquartile range (25th to 75th percentile). Each animal's individual data points are shown and color-coded to differentiate between males (dark blue) and females (pink)} with group outliers being outlined in black. *p ≤ 0.05.

The dietary interventions had the desired effects on the plasma Phe concentrations, in which the unrestricted (high Phe), semi Phe-restricted, and Phe-restricted diet were 2,160, 1,570, and 440% higher, respectively, than the 79 μmol/l in WT mice on AIN-93M diet (Figure 1E) [published in more detail by Van Vliet (26)].

### Behavioral Outcome

A clear phenotype was observed for exploratory behavior and motor performance, whereas we observed no phenotype on learning and memory outcomes. For the open field test we observed that all PKU animals, regardless of dietary treatment, spent less time in the center compared with the WT animals [Diet; *F*_(3,39)_ = 14.93, *p* < 0.001; all pairwise comparisons with WT, *p* < 0.001]. No differences between groups were found in learning and memory, although a significant interaction between sex and diet was found for the outcome of the SOR [Diet:sex; *F*_(3,36)_ = 3.88, *p* = 0.017]. A separate analysis of the males and females showed an insignificant trend toward an effect of diet in the male mice [*F*_(3,19)_ = 2.85, *p* = 0.065]. The lack of significant differences between the groups can possibly be explained by the observation that none of the groups performed significantly different from the chance level in both the novel object recognition [WT, *t*_(12)_ = 0.91, *p* = 0.190; Phe-restricted, *t*_(8)_ = 0.66, *p* = 0.264; semi Phe-restricted, *t*_(11)_ = 0.55, *p* = 0.296; PKU high Phe, *t*_(9)_ = 1.16, *p* = 0.138], and the spatial object recognition [WT, *t*_(12)_ = 0.37, *p* = 0.359; Phe-restricted, *t*_(8)_ = −0.76, *p* = 0.766; semi Phe-restricted, *t*_(10)_ = 0.19, *p* = 0.427; PKU high Phe, *t*_(8)_ = 1.00., *p* = 0.174], indicating that the task was too hard for the mice to master. For motor performance, we again observed a PKU phenotype for the percentage of good steps on the balance beam, regardless of dietary treatment [Diet; *F*_(3,34)_ = 7.77, *p* < 0.001; all comparisons with WT, *p* = 0.002]. All behavioral results are visualized in Figures 1A–D.

### Microbiome Richness and Evenness

**Figure 2:**
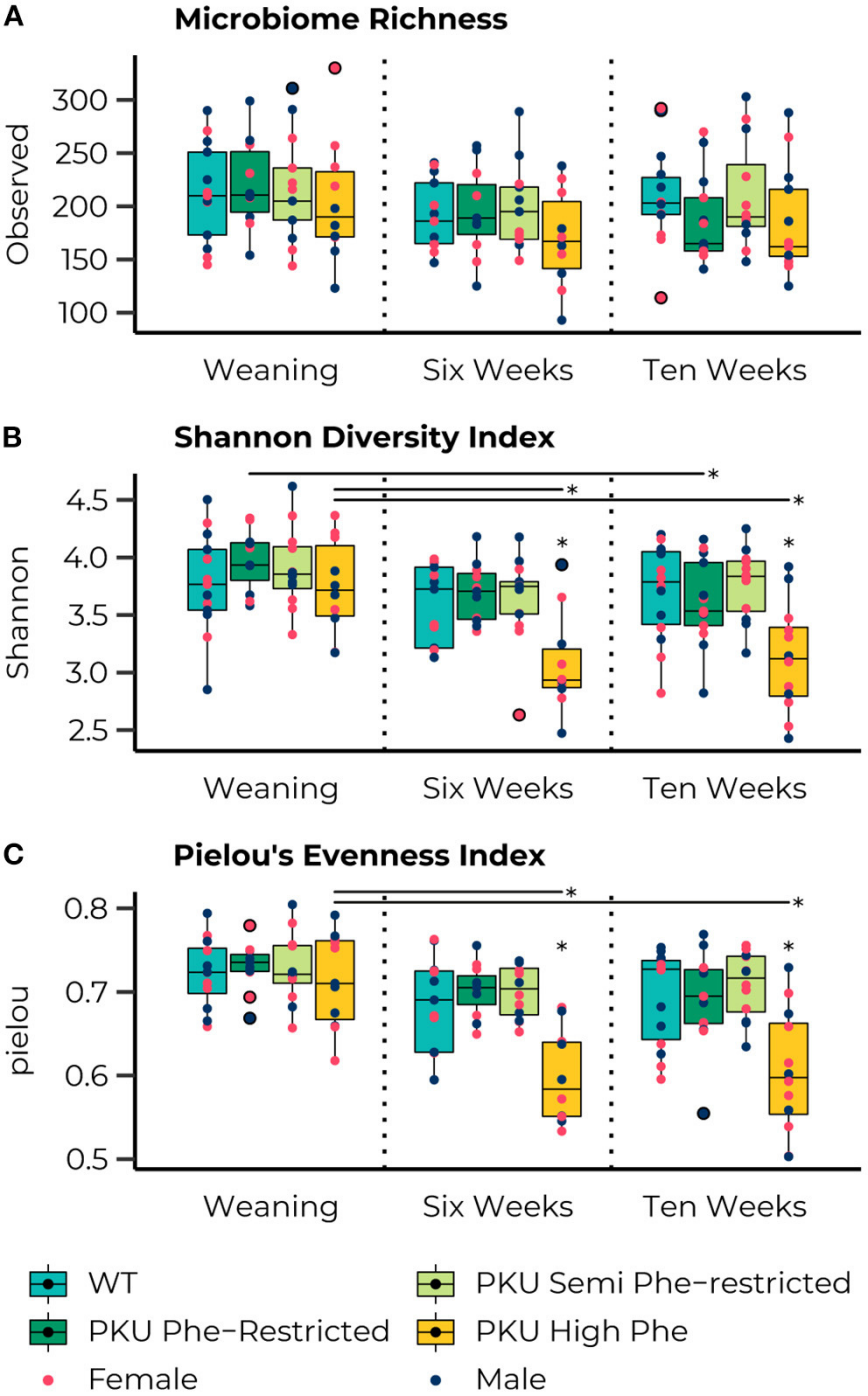
Boxplots representing the median (50th percentile) and interquartile range (25th to 75th percentile) of the number of observed amplicon sequence variants (ASVs) **(A)**, Shannon diversity index **(B)**, and Pielou's Evenness Index **(C)** at the three measured time points (start of diet after weaning and 6 and 10 weeks later) with individual data points showing the sex of the animal (females, pink; males, dark blue) and group outliers being outlined in black. The PKU on either a Phe-restricted (dark green), semi Phe-restricted (light-green), or unrestricted (high Phe; yellow) diet does not impact the alpha diversity in terms of the number of observed ASVs (Richness) compared with WT mice (blue). However, both the high Phe and Phe-restricted diet lead to a reduction in Shannon diversity over time [High Phe, *F*_(2,26)_=6.79, *p* = 0.004; Phe-restricted, *F*_(2,29)_=4.01, *p*= 0.029]. This change lead to significantly lower Shannon diversity in the PKU animals on high Phe after both 6 weeks (WT, *p* = 0.014; Phe-restricted, *p* = 0.014; semi Phe-restricted, *p* = 0.022) and 10 weeks (WT, *p* = 0.011; Phe-restricted, *p* = 0.014; semi Phe-restricted *p* = 0.009) of diet. Similarly, an effect of diet was observed in Pielou's evenness index at week 6 [*H*_(3)_ = 15.33, *p* = 0.002] and week 10 [*H*_(3)_ = 13.32, *p* = 0.004], with a clear time effect in the PKU high Phe group [*F*_(2,7)_ = 10.60, *p* = 0.000] leading to a lower evenness at both time points compared with the start of treatment (6 weeks, *p* = 0.002; 10 weeks, *p* = 0.002). At both time points, the PKU animals on high Phe showed a lower evenness compared to the WT animals and both forms of Phe-restriction (WT, *p* = 0.017 and *p* = 0.009; Phe-restricted, *p* = 0.002 and *p* = 0.022; semi Phe-restricted, *p* = 0.002 and *p* = 0.004, for week 6 and 10, respectively). *p ≤ 0.05.

The ASV richness remained stable over time, with no significant differences for each group across all time points, and no significant main or interaction effects were observed for sex (Figure 2A). At the start of the treatment (weaning), the Shannon Diversity index (Figure 2B) was similar across all groups. However, significant effects of time were observed in the high Phe [*F*_(2,26)_= 6.79, *p* = 0.004] and Phe-restricted [*F*_(2,29)_= 4.01, *p* = 0.029] PKU groups, both due to a change in the Shannon diversity after the start of the diet (PKU high Phe, *p* = 0.009 and *p* = 0.011; Phe-restricted, *p* = 0.08 and *p* = 0.020 for week 6 and 10, respectively). In the latter case, we observed a significant change 6 weeks after initiating the diet, remaining stable afterward. In the high Phe PKU group, we observed a significant effect [*H*_(3)_ = 11.38, *P* = 0.010] after 6 weeks of dietary treatment, revealing an extreme reduction in the Shannon Diversity index compared with all other groups (WT, *p* = 0.012; Phe-restricted, *p* = 0.009; semi Phe-restricted, *p* = 0.020) (Figure 2B*)*. This difference was still present at 10 weeks of treatment [*F*_(3,43)_ = 6.26, *p* = 0.001] (WT, *p* = 0.004; Phe-restricted, *p* = 0.012; semi Phe-restricted *p* = 0.002). Similar results were observed for the Simpson diversity index in which a decrease after the start of diet for the PKU high Phe [*F*_(2,29)_ = 4.75, *p* = 0.017] group (start to 6 weeks, *p* = 0.012; 10 weeks, *p* = 0.033), with a significant effect of diet after 6 weeks of treatment [*H*_(3)_ = 10.77, *p* = 0.013]. The *Post Hoc* analysis showed a lower Simpson diversity index for the high Phe PKU group compared with both degrees of Phe-Restriction (Phe-restricted, *p* = 0.012; semi Phe-restricted *p* = 0.009), although we observed no significant effect at 10 weeks of treatment [*H*_(3)_ = 6.25, *p* = 0.1] (Supplementary Figure 1A). For Pielou's evenness index, an effect of diet was observed starting from week 6 of treatment [*H*_(3)_ = 15.33, *p* = 0.002] and still present at week 10 [*H*_(3)_ = 13.32, *p* = 0.004], with a clear time effect in the PKU high Phe group [*F*_(2,27)_ = 10.60, *p* = 0.000] leading to a lower evenness at both time points compared with the start of treatment (6 weeks, *p* = 0.002; 10 weeks, *p* = 0.002) (Figure 2C). At both time points, the PKU animals on high Phe showed a lower evenness compared with the WT animals and both forms of Phe-restriction (WT, *p* = 0.017 and *p* = 0.009; Phe-restricted, *p* = 0.002 and *p* = 0.022; semi Phe-restricted, *p* = 0.002 and *p* = 0.004, for week 6 and 10, respectively). No significant differences were observed for Faith's phylogenetic diversity between dietary groups at the different time points. Still, a significant effect of time was observed in the high Phe PKU group [*H*_(2)_ = 6.13, *p* = 0.047], but this did not result in significant *post hoc* comparisons after *p*-value correction (Supplementary Figure 1B). No significant effects or interaction effects were observed for sex in any of the alpha-diversity measures.

### Community Structure

**Figure 3:**
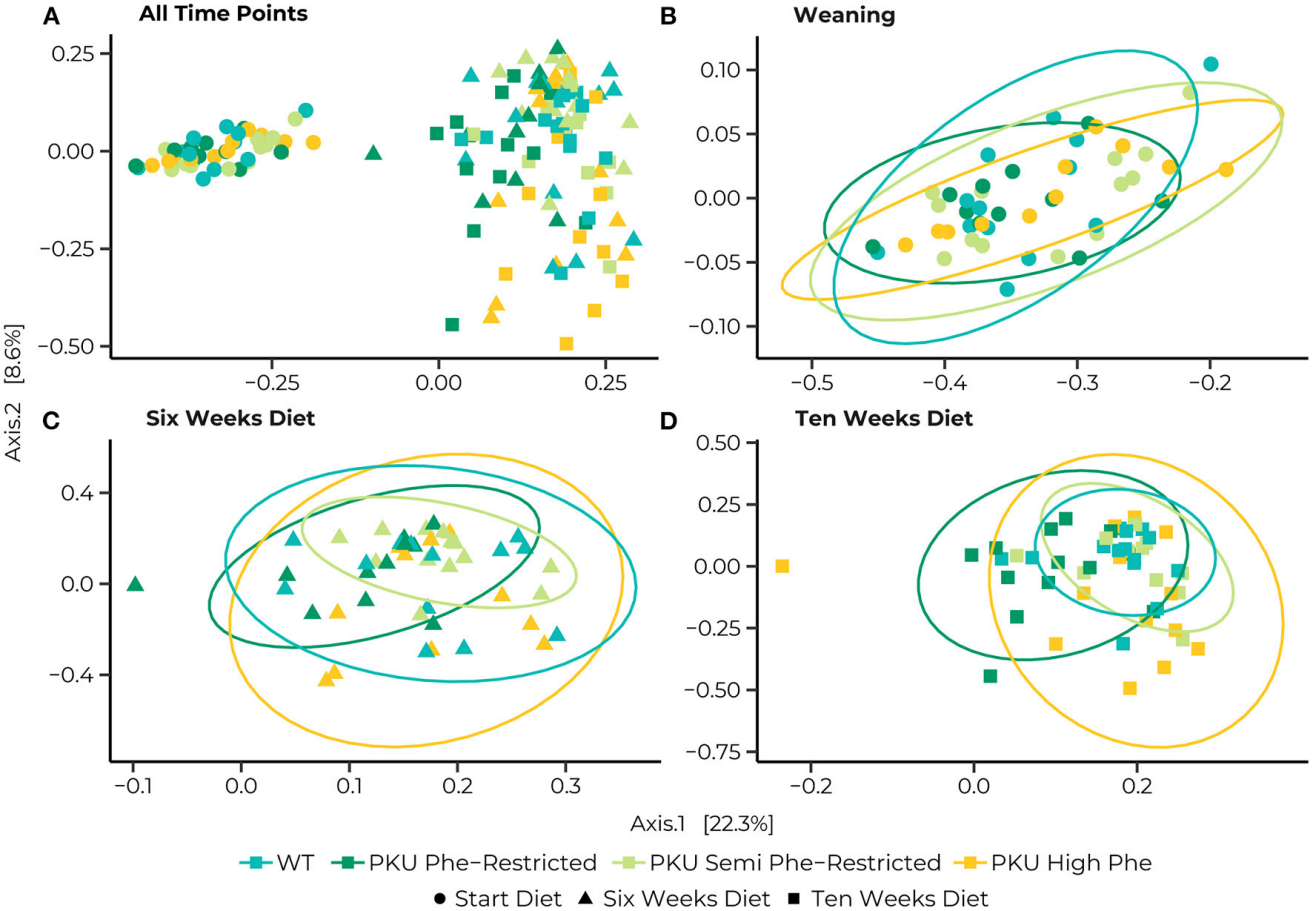
Principle coordinates analyses (PCoA) of Bray-Curtis distances for the entire dataset **(A)**, and subsets at the Start **(B)**, 6 weeks **(C)**, and 10 weeks **(D)** of the diet. Statistical ellipses are drawn at a 0.95 confidence level. A clear shift in community structure is observed from the start of the treatment (dots) to both 6 weeks (triangles) and 10 weeks (squares), with a statistical interaction between time point and dietary treatment [*F*_(6, 147)_ = 1.3852, *p* = 0.015]. Pairwise comparisons at the start of the diet show no differences between the WT (blue) and PKU animals on either a Phe-restricted (dark green), semi Phe-restricted (light green), or high Phe (yellow) diet. At 6 weeks, however, differences were observed between the Phe-restricted group and all other dietary treatments (WT, *p* = 0.009; semi Phe-restriction, *p* = 0.032; and PKU high Phe, *p* = 0.014), the PKU high Phe mice compared to the semi Phe-restricted treatment (*p* = 0.047). At 10 weeks of diet, all PKU animals show a significantly different community structure from the WT animals (Phe-restriction, *p* = 0.003; Semi Phe-restriction, *p* = 0.021; and PKU high Phe, *p* = 0.024). Between PKU groups, we observe that the semi Phe-restricted animals no longer differ significantly from either PKU high Phe (*p* = 0.102) or the Phe-restricted fed animals (p = 0.06). However, a significant difference remains between the Phe-restricted treated animals and the PKU animals on high Phe (0.015).

The PERMANOVA of the Bray-Curtis dissimilarities showed a significant interaction effect in bacterial communities between different time points and diets [*F*_(6, 147)_ = 1.39, *p* = 0.015] as well as effects of diet [*F*_(3,147)_ = 3.46, *p* = 0.001], and time point [*F*_(2,147)_ = 25.57, *p* = 0.035] by itself (Figure 3A). At the start of treatments, no differences were found between the different dietary groups. Still, we found that the bacterial communities significantly changed between time points for all dietary treatments (Figures 3B–D). After 6 weeks of treatment, differences were observed between the bacterial communities of the Phe-restricted group and all other dietary treatments (WT, *p* = 0.009; semi Phe-restriction, *p* = 0.032; and PKU high Phe, *p* = 0.014), the PKU high Phe mice compared to the semi Phe-restricted treatment (*p* = 0.047) (Figure 3C). The difference with the Phe-restricted treatment remained at 10 weeks, although this was no longer significantly different from the semi Phe-restricted animals (WT, *p* = 0.003; semi Phe-restriction, *p* = 0.06; and PKU high Phe, *p* = 0.024). Lastly, we observed a difference between the bacterial communities of the WT animals and bacterial communities of the semi Phe-restriction treated and control PKU mice (Semi Phe-restriction, *p* = 0.021; and PKU high Phe, *p* = 0.024), thus showing differences from all PKU groups (Figure 3D).

We examined the multivariate dispersion between groups. We observed no significant effects of dietary treatment [*F*_(3,144)_ = 0.635, *p* = 0.593], nor an interaction with time [*F*_(11, 136)_ = 1.123, *p* = 0.348]. We did however observe a significant effect of time [*F*_(2,145)_ = 8.0815, *p* = 0.001]. This effect was due to a difference between the overall dispersion at the last sampling point with the earlier time points (start vs. end, *p* = 0.001; 6 vs. 10 weeks, *p* = 0.033; start vs. 6 weeks = n.s.), suggesting that samples were more dispersed in their bacterial communities at the last sampling point (Supplementary Table 2), indicating a divergence in bacterial communities between dietary treatments over time.

### Taxonomic Differences

Visualization of relative abundance of taxonomic phyla, families, and genera for the different experimental groups for each time point can be found in Supplementary Figure 2. To determine the statistical differences in the observed abundances, pairwise comparisons between dietary groups were made at these taxonomic levels using DESeq2. At the start of the treatment (weaning), we observed one significant difference in abundance at each taxonomic level between the Phe-restricted and semi Phe-restricted animals, which was likely caused due to a difference in the *Mucispirillum* genus (*p* = 0.018), the *Deferribacteraceae* family (*p* = 0.008) and *Deferribacteres* phylum (*p* = 0.004).

**Figure 4:**
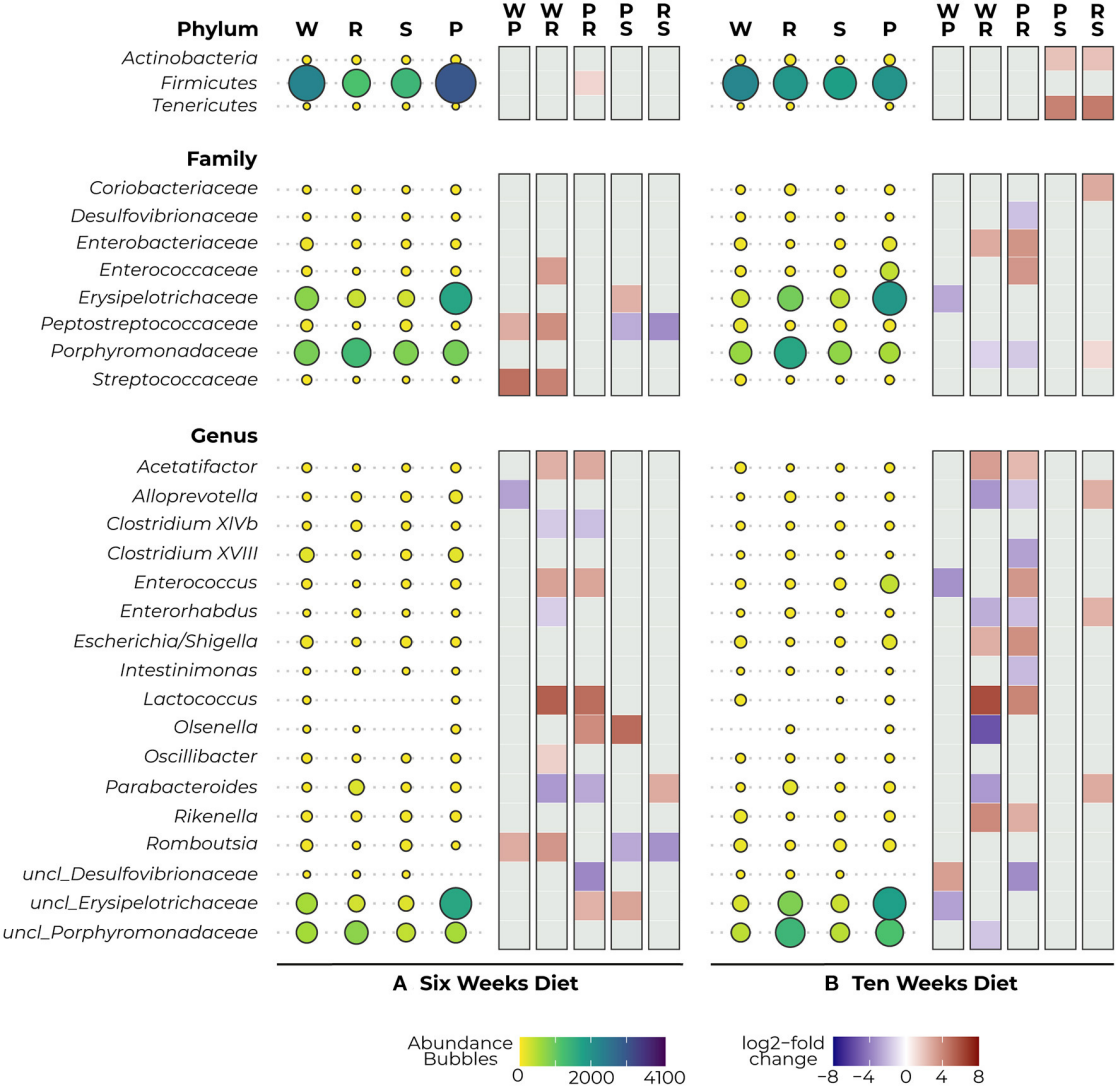
Mean abundance (per diet) and differential expression (between diets) of significantly different bacterial phyla (top), families (middle), and genera (bottom) as detected with DESeq2 analysis after 6 **(A)** and 10 **(B)** weeks diet. The bubbles represent the abundance of the taxa for each experimental group (WT, W; PKU Phe-restricted, R; PKU semi Phe-restricted, S; PKU high Phe, P), with bubble size and color representing abundance (small yellow = low abundance, large purple = high abundance). The heat plot shows differential abundance between diets in a log2-fold change as calculated by DESeq2, ranging between 8 (red) and −8 (blue), with a positive value indicating an increase in abundance of the first group of the contrast and a negative value indicating a decrease of this group (thus an increase in the lower group of the second group of the contrast).

After 6 weeks of diet, we observed a reduction in *Firmicutes* in the protein-restricted animals compared to the PKU animals on high Phe, which was significant for the more severe Phe-restriction (*p* = 0.012) (Figure 4A–*Phylum*). At the family level, we observed a decrease in the abundance of *Peptostreptococcaceae* (PKU high Phe, *p* = 0.017; Phe-restricted, *p* < 0.000) and *Streptococcaceae* (PKU high Phe, *p* < 0.000; Phe-restricted, *p* = 0.002) in both the PKU high Phe and Phe-restricted group compared with the WT animals, whereas the abundance of *Peptostreptococcaceae* seemed unchanged in the semi Phe-restricted group (PKU high Phe, *p* = 0.042; Phe-restricted, *p* < 0.000). Compared with the WT, we also found a reduction of *Enterococcaceae* in the Phe-restricted animals (*p* = 0.021), which was not seen in the other PKU groups. Additionally, we observed a higher abundance of *Erysipelotrichaceae* in PKU animals on high Phe compared with both forms of Phe-restriction, although this only reached statistical significance compared with the semi Phe-restricted group (Phe-restricted, *p* = 0.051; semi Phe-restricted, *p* = 0.006) (Figure 4A–*Family*). At the genus level, we observed two significant differences between the WT and the PKU animals on high Phe, with an increase of *Alloprevotella* (*p* < 0.001) and a decrease in *Romboutsia* (*p* < 0.001) in the PKU animals. The majority of the differences were observed between the high Phe treated animals (both WT and PKU) and the Phe-restricted animals, in which we saw a decrease in *Acetatifactor* (both genotypes, *p* = 0.001), *Enterococcus* (WT, *p* = 0.016; PKU, *p* = 0.034), *Lactococcus* (WT, *p* = 0.006; PKU, *p* = 0.025), *Olsenella* (PKU, *p* = 0.034), *Oscillibacter* (WT, *p* = 0.005), and *unclassified_Erysipelotrichaceae* (PKU, *p* = 0.008) in the Phe-restricted animals. Alternatively, increased abundances in this group were found for *Clostridium XlVb* (WT, *p* = 0.016; PKU, *p* = 0.008), *Enterorhabdus* (WT, *p* = 0.038), *Parabacteroides* (both genotypes, *p* < 0.000), and *unclassified_Desulfovibrionaceae* (PKU, *p* = 0.011). In the semi Phe-restricted group, we saw the same reduction in the *Olsenella* (*p* = 0.012) and *unclassified_Erysipelotricaceae* (*p* = 0.003) families compared to PKU on high Phe. Still, the increase of *Parabacteroides* that we found in the Phe-restricted group was not present on a semi-restricted diet (*p* = 0.000), and the increase in *unclassified_Desulfovibrionaceae* did not reach statistical significance (PKU high Phe, *p* = 0.053). Similar to the results of *Peptostreptococcaceae* at the family level, we observed that the decrease of *Romboutsia* in the PKU groups on high Phe and the Phe-restriction compared to the WT animals was again not present on a milder protein restriction (PKU high Phe, *p* = 0.036; PKU Phe-restricted, *p* = 0.002) (Figure 4A–*Genus*).

After 10 weeks of diet, we no longer observed a statistical difference in *Firmicutes* between the dietary groups; instead, we observed a large reduction in *Tenericutes* and a smaller reduction in *Actinobacteria* in animals that were treated with the semi Phe-restricted diet, which was significant compared to the other PKU animals (High Phe, both *p* = 0.031; Phe-restriction, both *p* = 0.027) (Figure 4B–*Phylum*). At the family level, the differences found at 6 weeks between WT and the PKU animals on high Phe and the Phe-restricted diet were no longer present, mainly due to increased abundances in the PKU groups. However, differences were found in abundance of *Enterobacteriaceae* (decrease; Phe-restricted, *p* = 0.046), *Erysipelotrichaceae* (increase; high Phe, *p* = 0.003), and *Porphyromonadaceae* (increase; Phe-restricted, *p* = 0.003) compared with WT animals. When comparing the PKU animals on high Phe and the Phe-restricted diet, we found differences in *Enterobacteriaceae* (*p* = 0.005) and *Enterococcaceae* (*p* = 0.009), which seem to be a result of lower abundances in the Phe-restricted group. On the other hand, we observed higher abundances of both *Desulfovibrionaceae* (*p* = 0.037) and *Porphyromonadaceae* (*p* = 0.000) in the PKU Phe-restricted group, the latter being also significantly different compared to the semi Phe-restricted group (*p* = 0.019). We also observed an increase in *Coriobacteriaceae* in the Phe-restricted group, but this only reached statistical significance compared to the semi Phe-restricted animals (*p* = 0.004) (Figure 4B–*Family*). Like the family level, we observed some shifts at the genus level, although this still resulted in the major differences found between the PKU animals on the Phe-restriction compared to the WT and PKU animals on high Phe. Similar differences as at 6 weeks of treatment were found for *Acetatifactor* (WT and PKU on high Phe, *p* = 0.001), *Enterococcus* (PKU, *p* = 0.003), *Enterorhabdus* (WT, *p* = 0.000), *Lactococcus* (WT, *p* = 0.000; PKU, *p* = 0.017), *Parabacteroides* (WT, *p* < 0.000) and *unclassified_Desulfovibrionaceae* (PKU, *p* = 0.002), whereas *Clostridium XlVb, Oscillibacter* and *Romboutsia* no longer showed a significant difference in abundance between diets. New differences were found between *Alloprevotella* (WT, *p* < 0.000; PKU, *p* = 0.038), caused by an increase in the Phe-restricted group not present in the semi-restricted group (*p* = 0.005) and *Clostridium XVIII* (PKU, *p* = 0.017) and *Intestinimonas* (PKU, *p* = 0.009), which seemed to be due to a decrease of this genus in the animals treated with High Phe. Additionally, we observed a reduction of *Escherichia/Shigella* in both Phe-restricted groups, which reached statistical significance for the full restriction (WT, *p* = 0.035; PKU, *p* = 0.002). Contrary to week 6, we observed that the *Olsenella* genus was now more abundant in the Phe-restricted group (WT, *p* = 0.032) and appeared to be low to not abundant anymore in the WT and semi Phe-restricted animals. Lastly, we observed a significant decrease in *Rikenella* (WT, *p* = 0.000; PKU, *p* = 0.014) and an increase in both *unclassified_Porphyromonadaceae* (WT, *p* = 0.026). Between the WT animals and PKU animals on high Phe, we only observed three differences, which were an increase in the PKU group for *Enterococcus* (*p* = 0.017) and *unclassified_Erysipelotrichaceae* (*p* = 0.015), and a decrease in *unclassified_Desulfovibrionaceae* (*p* = 0.015). Interestingly, no statistical differences were observed at both family and genera levels between the PKU group on the high Phe and the semi Phe-restricted group. However, differences in average abundance can be seen visually (Figure 4B–*Genus*). All results are visualized in Figure 4, showing both the abundances and log2-fold changes of comparisons between dietary groups.

### Correlations With Plasma Phe Levels and Behavior

**Figure 5:**
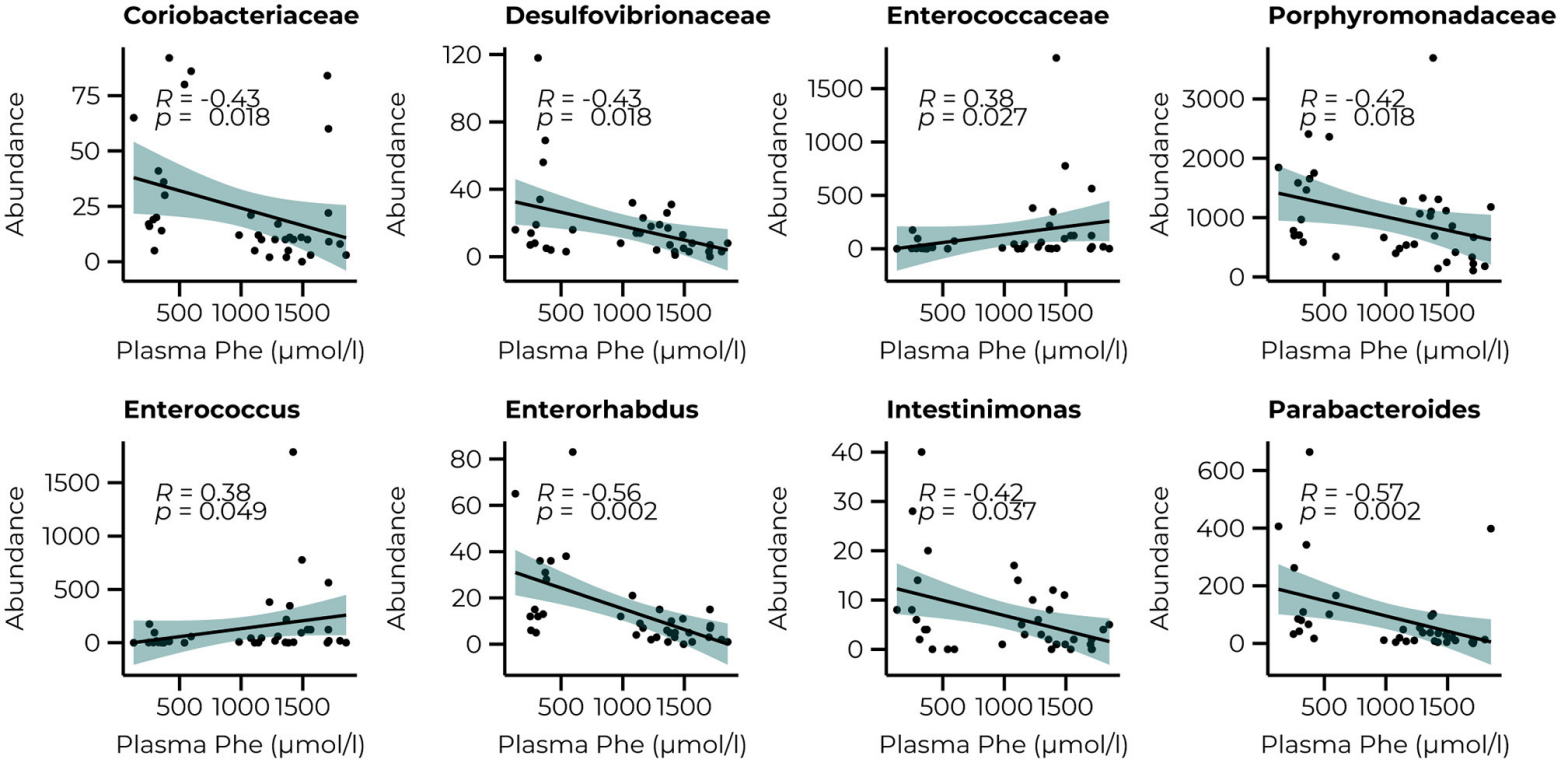
Significant correlations between the abundance of bacterial families (*Coriobacteriaceae, Desulfovibrionaceae, Enterococcaceae*, and *Porphyromonadaceae*) and genera (*Enterococcus, Enterorhabdus, Intestinimonas*, and *Parabacteroides*) after 10 weeks of diet and plasma Phe levels.

To link the bacterial taxonomy to the protein-restricted diet, we correlated the count data of the significant families and genera of the PKU animals with the plasma Phe levels we collected at termination (10 weeks) as an outcome measure of the efficacy of the dietary Phe-restriction (Figure 5). At the family level, we observed a significant negative relationship with *Coriobacteriaceae, Desulfovibrionaceae*, and Porphyromonadaceae (all comparisons, *R* = −0.43, *p* = 0.018, and a positive relationship with *Enterococcaceae* (*R* = 0.38, *p* = 0.027). At the genus level, we again observed mostly negative correlations (3 out of 4), specifically for *Enterorhabdus* (R = −0.56, *p* = 0.002), *Intestinimonas* (*R* = −0.42, *p* = 0.037), *Parabacteroides* (*R* = −0.57, *p* = 0.002), and one positive relationship with *Enterococcus* (*R* = 0.38, *p* = 0.049). A trend toward a positive relationship with *Acetatifactor* was also observed, but this did not reach statistical significance (*R* = 0.37, *p* = 0.053). All correlations, estimates, and unadjusted and adjusted *p*-values are summarized in Supplementary Table 3.

**Figure 6:**
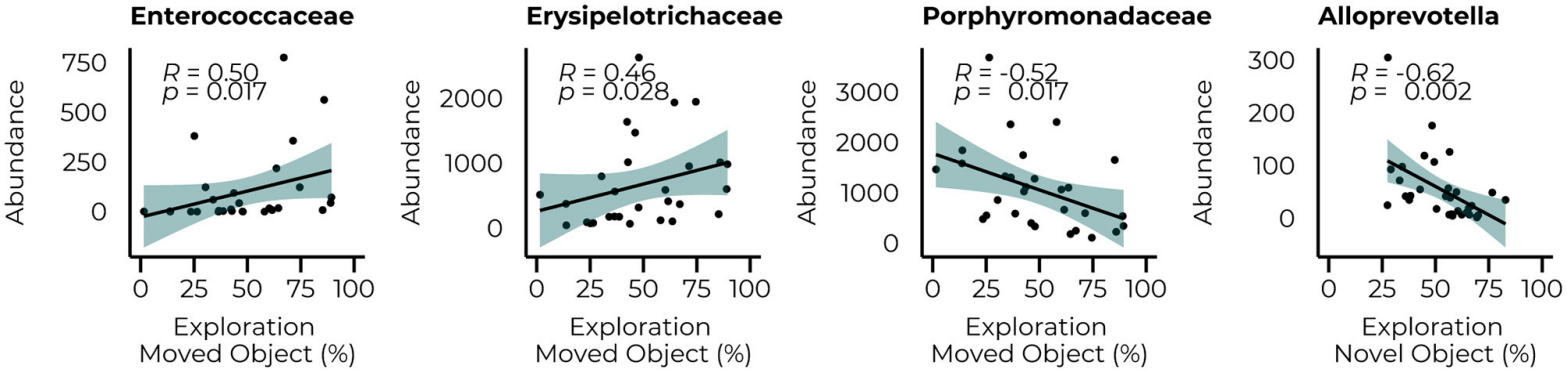
Significant correlations between the abundance of bacterial families (*Enterococcaceae, Erysipelotricaceae*, and *Porphyromonadaceae*) 10 weeks of diet and the *Alloprovotella* genus after 6 weeks of the diet with the behavioral outcome.

The same analysis was repeated for the behavioral parameters for both the count data at 6 and 10 weeks of treatment (Figure 6) to examine whether performance in the behavioral tasks could be related to the abundance of taxa that were significantly different between groups at their relative time points. Although our animals could not master the learning tasks (NOR and SOR) on a group level, we observed large individual variation between the animals within the same group, allowing us to examine whether specific bacterial taxa were related to better performance on these behaviors. Limited relationships were observed with the microbiome data at 6 weeks but included a negative relationship with the *Alloprevotella* genus (*R* = −0.62, *p* = 0.002). With the inclusion of the WT animals, a negative relationship between the *Streptococcaceae (R* = 0.43*, p* = 0.007*)* family and the time spent in the center of the open field was observed and still led to a significant correlation with the *Alloprevotella* genus (*R* = −0.47, *p* = 0.013) Correlations with microbiome data of week 10 of treatment resulted in several relationships with the performance in the SOR (time spent exploring the moving object). At the family level, we observed positive relationships between the *Enterecoccaceae* (*R* = 0.50, *p* = 0.017) and *Erysipelotricaceae* (*R* = 0.46, *p* = 0.028) families and a negative relationship with the *Porphyromonadaceae* family (*R* = −0.52, *p* = 0.017). The correlations between the PKU animals and taxonomic abundance are shown in Supplementary Table 3; correlations with the inclusion of the WT animals are presented in Supplementary Table 4.

## Discussion

The consequences of the restricted dietary treatment of patients with PKU are often associated with heterogenic neurocognitive, psychosocial, and metabolic responses. Changes in the gut microbiome of patients with PKU could be related to this heterogeneity, given the importance of diet in gut microbiome regulation and the role the gut microbiome plays in the gut-brain axis. In this study, we tried to disentangle these components–gut microbiome, diet, and behavior–by experimentally testing the response of PKU mutant mice to diets with different Phe contents. Below we discuss these results in the context of how the microbiome composition changes as a result of PKU, the effect of Phe-restriction, and the distinct bacterial taxa that are associated with increased plasma Phe levels. The results could aid the development of new therapeutic approaches for the improved treatment of PKU.

### PKU Disorder Leads to Altered gut Microbiome Composition, Which Can Be Partially Restored With Semi—Phe Restricted Diets

**Figure 7:**
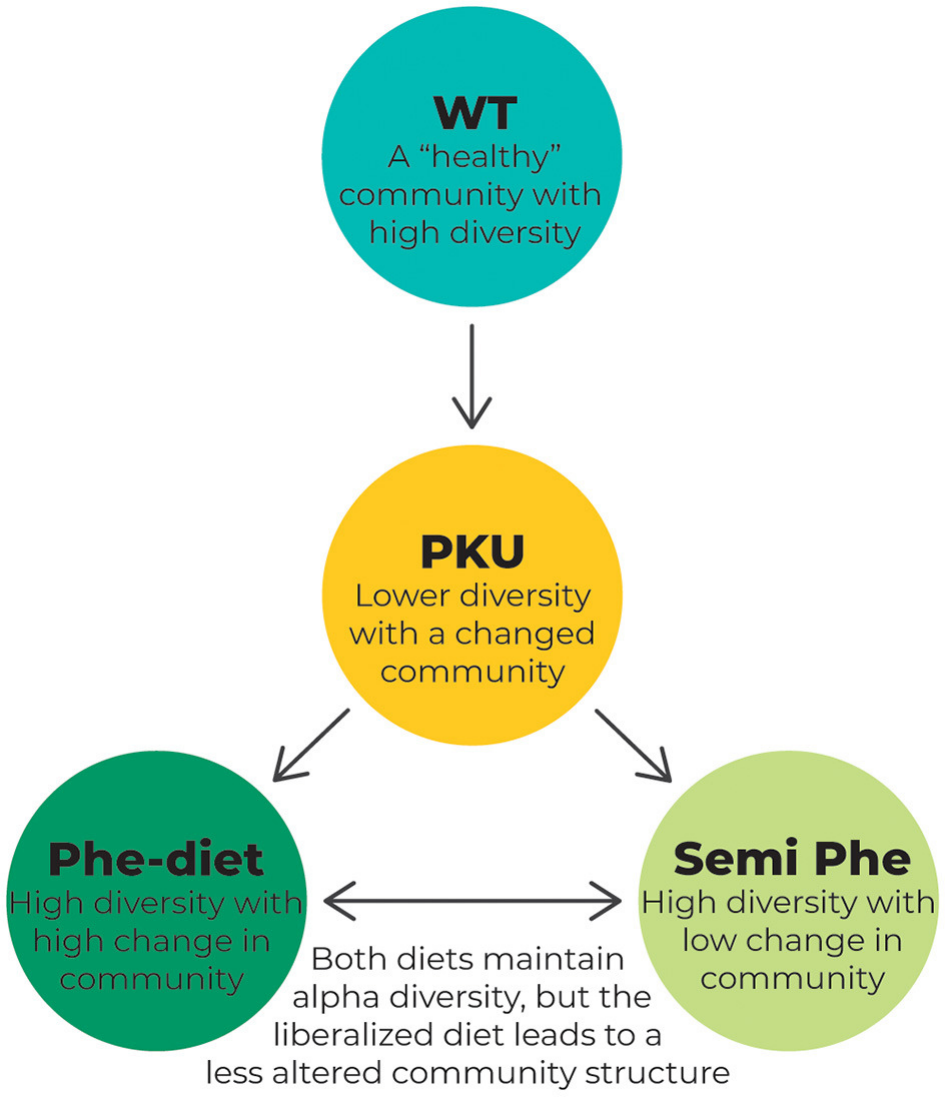
Schematic overview of the changes in the microbiome composition as a result of PKU and/or dietary treatment. The WT animals represent a microbiome that is not affected by the disease. This “healthy” microbiome is thought to show high diversity, leading to a resilient microbial community. In PKU, however, this diversity is challenged by the metabolic disorder causing high Phe levels. This leads to a lower diversity as measured by the Shannon diversity index and Pielou's evenness and a change in overall community composition, as measured by PCoA using the Bray-Curtis distance. Treating the PKU animals with either a Phe-restricted or a semi Phe-restricted diet prevents the loss of alpha diversity caused by PKU. Still, it shifts the bacterial community composition even further from the bacterial community of the WT animals than the PKU condition itself. This change in community composition is more prominent in the case of full Phe-restriction, showing the biggest distance from the WT microbiome when compared with both the unrestricted and semi Phe-restricted diet.

The results of our study showed that untreated PKU greatly impacted the microbiome alpha diversity in terms of the Shannon Diversity Index and that PKU influenced gut bacterial community structure (beta diversity) regardless of treatment in the PKU mouse model (see Figure 7 for a schematic overview). These results are similar to previously reported findings in both humans (21, 24, 25) and mice (22), in which PKU individuals on a Phe-restricted diet have, or tend to have, a less diverse and even bacterial community that differs from either non-PKU or mild hyperphenylalaninemia subjects, although inconsistencies between studies exist. Remarkably, Phe-restriction leads to the largest change in community structure, which seems to be lessened by allowing some intake of natural protein (semi Phe-restriction). It is interesting to note that these differences seem to develop faster depending on the severity of Phe-restriction; after 6 weeks of diet, the PKU Phe-restricted animals already showed a different community structure compared with the other PKU and WT animals, whereas at the same time point, the semi Phe-restricted animals did not yet significantly differ from WT. After 10 weeks, however, all PKU animals had significantly different microbial communities compared with the WT animals. There was a difference between the PKU animals on high Phe compared with both forms of Phe-restriction. This suggests that although diet influences the microbiome composition strongly (i.e., the taxonomic differences between the WT animals and PKU animals on the high Phe diet seem to be less pronounced than those between the WT animals and Phe-restricted animals), the PKU condition still affects the community structure. However, in terms of alpha diversity, the semi Phe-restricted animals seem to be most successful in maintaining a rich and even microbiome in the context of PKU compared to the WT mice. A high(er) diversity leads to a biotic barrier, preventing the establishment and growth of (non-)pathogenic invaders from the environment. These disturbances are common and can lead to significant shifts in the community due to microbial competition if the resident microbial community is not able to outcompete the invader and return to the original (healthy) state (16, 43, 44). Therefore, from an ecological perspective, the semi Phe-restricted diet could potentially lead to an altered but more stable and resilient gut microbiome than the high Phe or Phe-restricted diets.

Of note, although our data suggest that PKU is the largest contributor to changes in alpha diversity, these changes were not evident at the start of treatment, the point up until they had been housed with their heterozygous (HTZ) mother and mixed genotype (WT, HTZ, PKU) siblings. Therefore, our data implies that either the proximity of diverse microbiomes (and potentially maternal nutrition) can prevent the effects of PKU on the microbiome regardless of high Phe levels or that these differences develop at a later time frame. It has been shown that living together has a higher impact than genetics on microbiome composition, even in humans (45). Additionally, Dill-McFarland et al. (45) also showed that people living alone have less diverse microbiomes than married individuals, suggesting that the differences are not only explained by nutritional habits. As mice are coprophagous, these effects of living together could be amplified by the recurrent inoculation of healthy microbes in social situations (46). If this is indeed the case, microbiome transplantations could potentially successfully establish a rich and diverse microbiome in PKU.

### Different Levels of Dietary Phe-Restriction Select for Distinct Bacterial Taxa, Which Can Partially Explain PKU Behavior

Looking at the specific members of the bacterial communities, previous studies have shown shifts in abundance at the phylum level. The most pronounced decrease in *Bacteroidetes, Firmicutes*, and *Verrucomicrobia* in children with PKU (±5 years) in the study of De Oliveira et al. (21). Although we did not observe any significant differences for any of these phyla between our Phe-restricted and WT animals, we did observe a tendency for a reduction in *Firmicutes* in our Phe-restricted animals. While there seems to be a general trend in the studies that show a decrease in *Bacteroidetes* (21, 24) in PKU subjects, remarkably, we observe higher base mean levels in the PKU Phe-restricted animals compared with our other groups, which however seems to be comparable to the new data provided by Mancilla et al. (25) which shows an increase of *Bacteroidetes* in the adult PKU microbiome. Similarly, the families that changed in response to treated PKU differ between studies, with substantial decreases in *Clostridiaceae, Erysipelotricaceae*, and *Lachnospiraceae* (21), *Veillonellaceae* and *Ruminococcaceae* (24), and *Lactococcus* in our study. Inconsistencies include an increase in *Lachnospiraceae* observed by *Bassanini*, but not by De Oliveira et al. (21), suggesting this is Phe related in young children with PKU. Lastly, we observed a decrease in *Peptostreptococcaceae*, contrary to the findings of De Oliveira et al. (21). Interestingly, we also show a variation between time points: although insignificant, after 6 weeks, lower base mean levels of the *Erysipelotrichaceae* family and the *unclassified_Erysipelotrichaceae* genus were observed in PKU Phe-restricted animals compared with WT, whereas after 10 weeks, the opposite was shown. Inconsistencies between these results can have numerous reasons, including the age or species of the subjects, environmental availability of microbes, and methodological differences (47, 48). Indeed, the recently published data from Mancilla et al. (25) show that the adult PKU microbiome differs from that of children, which could explain the differences observed between studies (25). At the genus level, there is considerable variability between studies as to whether particular bacteria are significantly abundant or not in response to PKU and/or the diet. However, these studies do show a consistency in the direction of change (i.e., an increase or decrease in abundance) for the same bacterial genera suggesting that these differences might be specific responses to PKU or the diet, regardless of age or host species. For example, both Mancilla et al. and De Oliveira et al. show an increase in *Akkermansia* in treated patients with PKU. Although we did not observe any significant changes, we did observe a vast increase at 6 weeks in both treated and untreated PKU animals, indicating that high variability could play a role in discrepancies between studies. Notably, abundances of several genera, including *Bacteroides, Blautia, Lactococcus, Oscillibacter, Romboutsia, Ruminococcus, and Veillonella*, remain inconsistent between studies and causes other than PKU/diet could contribute to their abundance.

Despite the differences between the human and murine gut-microbiome, our results provide insights into how different Phe contents in the diet, leading to altered blood Phe levels, relate to distinct bacterial taxa present in the microbial community. We observed that PKU itself, on a high Phe diet, is associated with an increase in *Firmicutes* after 6 weeks of a diet that normalizes after 10 weeks of treatment. At the family and genus level, we observe a reduction in *Peptostreptococcaceae, Streptococcaceae*, and *Romboutsia* as opposed to an increase in *Alloprevotella* at 6 weeks and *Erysipelotrichaceae* and *Enterococcus* at 10 weeks. Interestingly, four relationships were found with plasma Phe levels out of the eight families that showed differential abundances between diets. Three of them led to lower abundances under higher Phe levels. Out of these four relationships, we observed that *Enterococcaceae* and *Porphyromonadaceae* are related to hippocampal-dependent memory in the spatial location object tests in favor of better performance under high Phe levels. Of note, although we do not observe a (significant) correlation with *Erysipelotrichaceae* and Phe levels, this family is present in high abundances in both treated and untreated PKU and is positively related to the performance in spatial memory. This relationship is supported by a positive correlation between abundances of the same family with Y-maze performance in a study by Sanguinetti et al. (49). Overall, our observations of relationships between microbiome and behavior could suggest that there might be adaptive mechanisms to prevent brain damage as a result of high Phe in untreated conditions and potentially explain why the behavioral testing in untreated PKU mice shows inconsistencies and a much milder phenotype than expected based the severe symptoms of untreated PKU in humans, as also reported in the current study.

At the genus level, we observed minimal differences in the abundance of individual bacterial taxa between the WT and PKU animals on high Phe, even though these differences were present at the community level (alpha and beta diversities). This could suggest that the changes in bacterial abundance per genera might be more subtle and/or show more variation than the Phe-restriction treated animals. For the selected genera, we established four relationships with Phe levels, of which the majority were negative (*Enterorhabdus, Intestinimonas*, and *Parabacteroides*), except for Enterococcus. Contrary to what we observed at the family level, none of these genera were related to behavioral outcomes. However, we observed that the abundance of the *Alloprevotella* genus at 6 weeks was associated with the novel object recognition test outcome, at the time point at which we see a significant increase in this genus in the PKU animals on the high Phe diet. Although the microbiome is a community of interacting members that affect host physiology and behavior, these correlations could indicate that some members directly affect brain functioning. On the other hand, there is a possibility that these bacteria have the potential of canceling each other's function, thereby contributing to the very heterogenic outcome/phenotype observed in both PKU mice and humans.

### Potential Microbiome-Based Therapeutic Strategies to PKU Disorder

While our study was exploratory, our results can be used as the starting point for future research into the development of probiotics to supplement dietary restriction in PKU treatment. Ideally, the presence and abundance of the chosen strains should be little affected by fluctuating Phe levels and should additionally show a positive relationship with cognitive behavior. We identified several bacterial taxa that adhere to this. Based on our results, we consider it would be worthwhile to focus the research on members from the *Enterococcaceae* and *Erysipelotricaceae* families, as they were in high abundance under high(er) Phe conditions and additionally showed a positive relationship with hippocampal-dependent memory in mice. In particular, research on the genera *Enterococcus, unclassified_Erysipelotricaceae*, and *Alloprevotella* should be conducted to further clarify their association with cognitive functioning and whether there is a potential to use these taxa in probiotic supplementation for PKU. In addition, taxa such as *Clostridium XVII, Escherichia/Shigella*, and *Lactococcus*, which were either more or exclusively abundant in the microbiome of high Phe animals relative to those of Phe-restricted animals, show potential for improvements not directly linked to behavior, such as an ability to increase diversity and resilience of the microbiome under high Phe levels.

It should be noted that literature on the effects of specific families or genera is often without consensus on their beneficial or detrimental impact on health. This could have several reasons, including that these effects occur primarily at the species level (i.e., families and genera could include both beneficial and disadvantageous microbial members), but also because the microbiome functions as one ecological community, in which bacteria might play different roles depending on their specific environment. For example, members of the *Entereococcaceae* family have been associated with disease, but others are already used as probiotics (50). Moreover, increased abundance of *Erysipelotricaceae* has been reported in patients with autism Specter disorder (51) and inflammation models (52), but otherwise is lowered in depression and Parkinson's disease (53, 54) and to be positively correlated with cognitive function (49). For *Erysipelotricaceae*, research also shows that it responds differently to diet or host health phenotypes, indicating that alterations in abundances can have a negative or beneficial impact depending on the disease (55). Among our genera of interest, we find genera that are already used in the food industry (*Lactococcus*) or as probiotics (*Enterococcus*) (56). Interestingly, the *Alloprevotella* genus has been associated with the production of short-chain fatty acid and amino acid metabolism in specific pathogen-free mice (57). Short-chain fatty acids (SCFAs) include acetate, propionate, and butyrate and are mainly produced by fermenting dietary fiber but can also be produced from amino acid metabolism. SCFAs are thought to play a crucial role in the functioning of the microbiome-gut-brain axis [for a review on the role of SCFAs in gut-brain communication, see (58)], making these genera interesting candidates to improve cognitive outcomes potentially.

Due to the lack of knowledge about microbial functioning and the inconsistency in the function of specific bacterial genera, the importance of isolating and identifying particular species for the development of microbiome-based therapeutic strategies should be emphasized. We have previously argued that for successful probiotic supplementation, general concepts of community dynamics (i.e., the potential interactions within the gut microbiome) and microbial invasions and colonization should be taken into consideration during the development of microbiome-based therapeutic strategies to increase the treatment effectiveness and safety (44). Isolation of the specific species will allow the determination of their metabolic potential and reinoculation of these species to examine their effects specifically in the context of PKU-related alterations in the microbiome composition. Ideally, these species could include members capable of utilizing and degrading Phe, thereby lowering Phe on the microbiome level if present in large but stable numbers. Using microbial-based therapeutic strategies in addition to the Phe-restricted diet can potentially improve the metabolic and behavioral outcome in treated patients with PKU by increasing the microbiome diversity and resilience while simultaneously allowing a more liberalized Phe-restriction by utilizing microbial metabolism.

## Conclusion

This study set out to examine whether the differences found in the microbiome composition of PKU patients compared with healthy controls were due to the dietary treatment, the disease itself, or an interaction between the two. Our results showed that both the PKU condition and its treatment affected the microbiome composition in the PKU mouse model. What is especially interesting is that lowering Phe-restrictions in the diet seems to be as effective as Phe-restriction in preventing PKU-related loss in the microbiome Shannon diversity and can potentially even be beneficial for the abundance of specific bacterial taxa and cognition (Figure 7). If translatable to humans, this could suggest that (adult) individuals who temporarily do not adhere to the restrictive diet might not experience additional deficits due to a change in the microbiome composition. Nevertheless, as the Phe-restricted diet is currently the most commonly available and effective treatment for PKU patients, it is imperative to understand its effects on the microbiome and subsequently host metabolism and behavior to optimize overall disease outcomes. Although our study suggests that Phe-restriction can prevent the PKU-related loss of alpha diversity in mice, we have observed that the Phe-restricted diet leads to significant shifts in microbiome composition, which could impact the functional diversity of the microbiome. This functional diversity determines the metabolites that remain to interact with the physiology of the host. Therefore, microbial-based strategies, including pre-and probiotics, could potentially be beneficial to the treatment of PKU by increasing the microbial diversity and making the microbiome more resilient. Having a more stable and resilient microbiome allows the gut to withstand shifts and prevent changes in functional diversity.

## Limitations and Future Perspectives

Although this study clearly showed that both the PKU condition and the diet influence the PKU microbiome, several limitations should be discussed. First of all, the translational value of our data should be interpreted with caution as there are many differences between mice and humans, including the anatomical differences of the gastrointestinal tract, metabolic turnover rate, and preferred food sources (herbivores vs. omnivores) (59). These factors can affect the phylogenetic composition of the bacterial communities. Although it has been shown that the human and murine microbiome can be considered similar at its core, the abundance of specific taxa is quantitatively different between the two species (60, 61). Moreover, studies have shown that the different segments of the gastrointestinal tract are not only specialized in regulating complexes but also harbor regional microbiota due to selection pressures created in that specific environment (62). These complex interactions between the host and the microbiome require more research to fully understand the community dynamics and organizational structure of the regional microbial communities. Lastly, although 16S rRNA amplicon sequencing is a great exploratory tool to determine differences in microbial communities, it is limited in the information it can provide about the functionality of the microbiome for the host. To improve our understanding of the functional consequences of the PKU microbiome, future research should include metagenomic and metatranscriptomic analyses.

In our study, we examined the relationships between Phe levels and behavior in our mouse model. Lowering the Phe content in the diet requires alterations in total protein content and diet composition in the ratio between intact protein and added amino acids. Moreover, Phe can (re)enter the gut by entero-recirculation, specifically in the small intestine, regardless of the PKU condition (63, 64). It has been suggested that this enterorecirculation leads to a reservoir of Phe, although more research is needed to fully understand the mechanistic and functional basis of this process. More research could give an insight into the fate of dietary Phe (restriction) in the gut and how this directly influences the microbiome composition. Additionally, amino acid profiles are likely to alter beyond just Phe levels due to the complex metabolic interactions in PKU and the competitive transport of large neutral amino acids over the blood-brain-barrier and gut-blood-barrier. We reason that in treating PKU, success is monitored by measuring the plasma Phe levels and that, although other minor differences between dietary treatments exist, the largest contributor is most likely the difference in Phe. Future research could include other aspects of amino acid and neurotransmitter metabolism to examine a wide range of potential relationships. As both the metabolic alterations in treated PKU and the microbiome community dynamics are complex, this study is just a first step in gaining more insight into the interplay between these complex processes.

## Data Availability Statement

The data presented in the study are deposited in the European Nucleotide Archive (ENA) repository, accession number PRJEB46382.

## Ethics Statement

The animal study was reviewed and approved by Institutional Animal Care and Use Committee of the University of Groningen.

## Author Contributions

EG, DV, FS, and EZ designed the study. EG performed breeding, animal care, sampling, and behavioral testing. EG, SV, JF, and EZ made the analysis and interpretation of the behavioral and microbiome data. EG drafted a first version of the manuscript and critically revised by DV, SV, FS, JF, and EZ. All authors contributed to the article and approved the submitted version.

## Funding

This research was funded by a research grant from the National PKU Alliance (USA) and supported by the Adaptive Life program of the Groningen Institute for Evolutionary Life Sciences (GELIFES).

## Conflict of Interest

The authors declare that the research was conducted in the absence of any commercial or financial relationships that could be construed as a potential conflict of interest.

## Publisher's Note

All claims expressed in this article are solely those of the authors and do not necessarily represent those of their affiliated organizations, or those of the publisher, the editors and the reviewers. Any product that may be evaluated in this article, or claim that may be made by its manufacturer, is not guaranteed or endorsed by the publisher.

## Supplementary Material

Supplementary Table 1 Nutritional and Amino acid content of the experimental diets.

Supplementary Table 2 Multivariate dispersion.

Supplementary Table 3 BaseMean of bacterial families and genera per diet and correlation analyses Phe-levels and behavior.

Supplementary Table 4 Correlations Phe-levels and behavior with both WT and PKU animals included.

Supplementary Figure 1 Simpson Diversity Index and Phylogenetic Diversity.

Supplementary Figure 2 Average relative abundance of bacterial phyla, families and genera.
