# Supplementary figures and images for: Gut-Microbiome Composition in Response to Phenylketonuria Depends on Dietary Phenylalanine in BTBR Pahenu2 Mice
Source: Front Nutr. 2022 Jan 4;8:735366. doi: 10.3389/fnut.2021.735366 (PMC8763796; doi:10.3389/fnut.2021.735366)

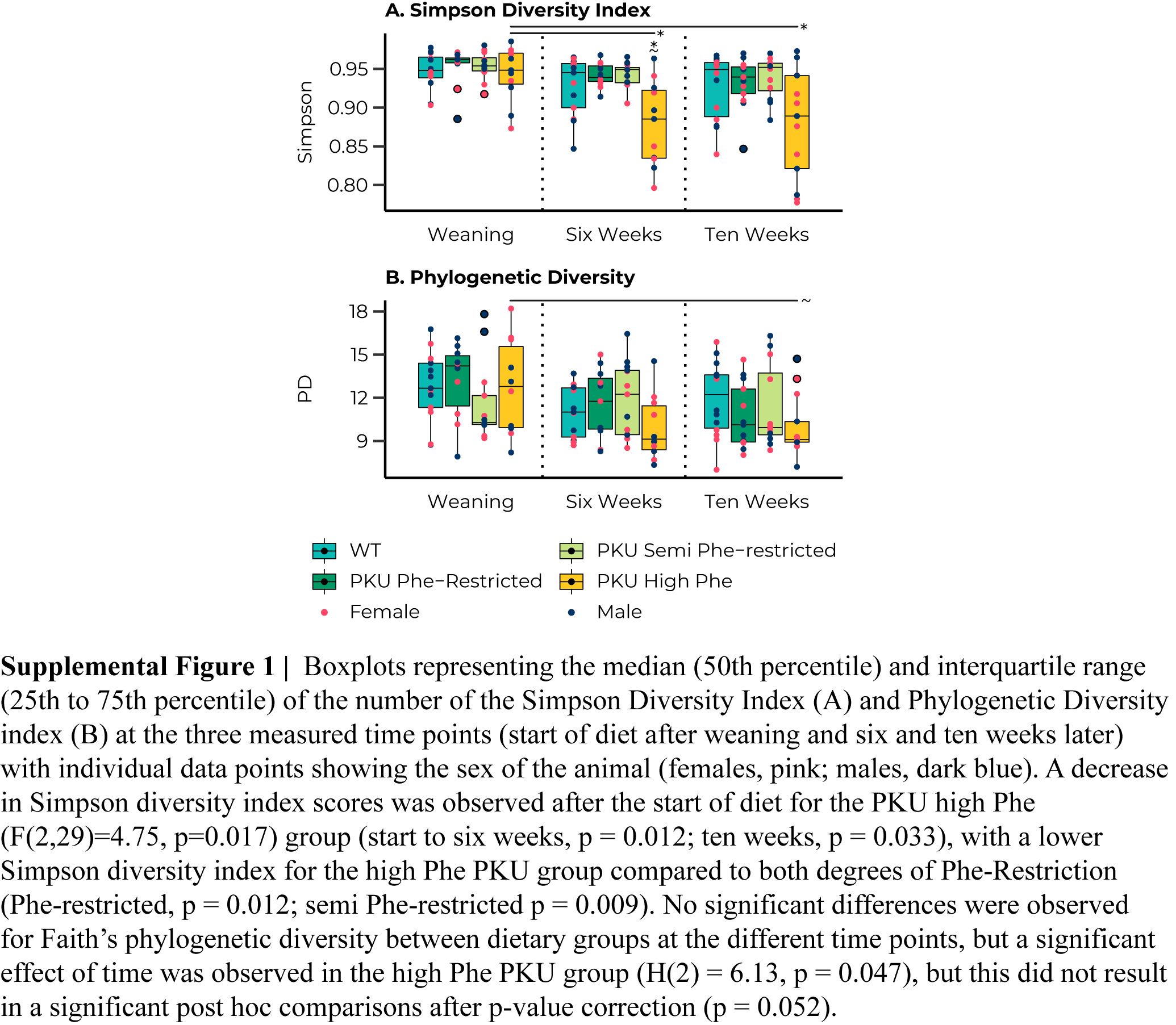

Supplement: Supplementary Figure 1 — Simpson Diversity Index and Phylogenetic Diversity. [file Image_1.TIF]

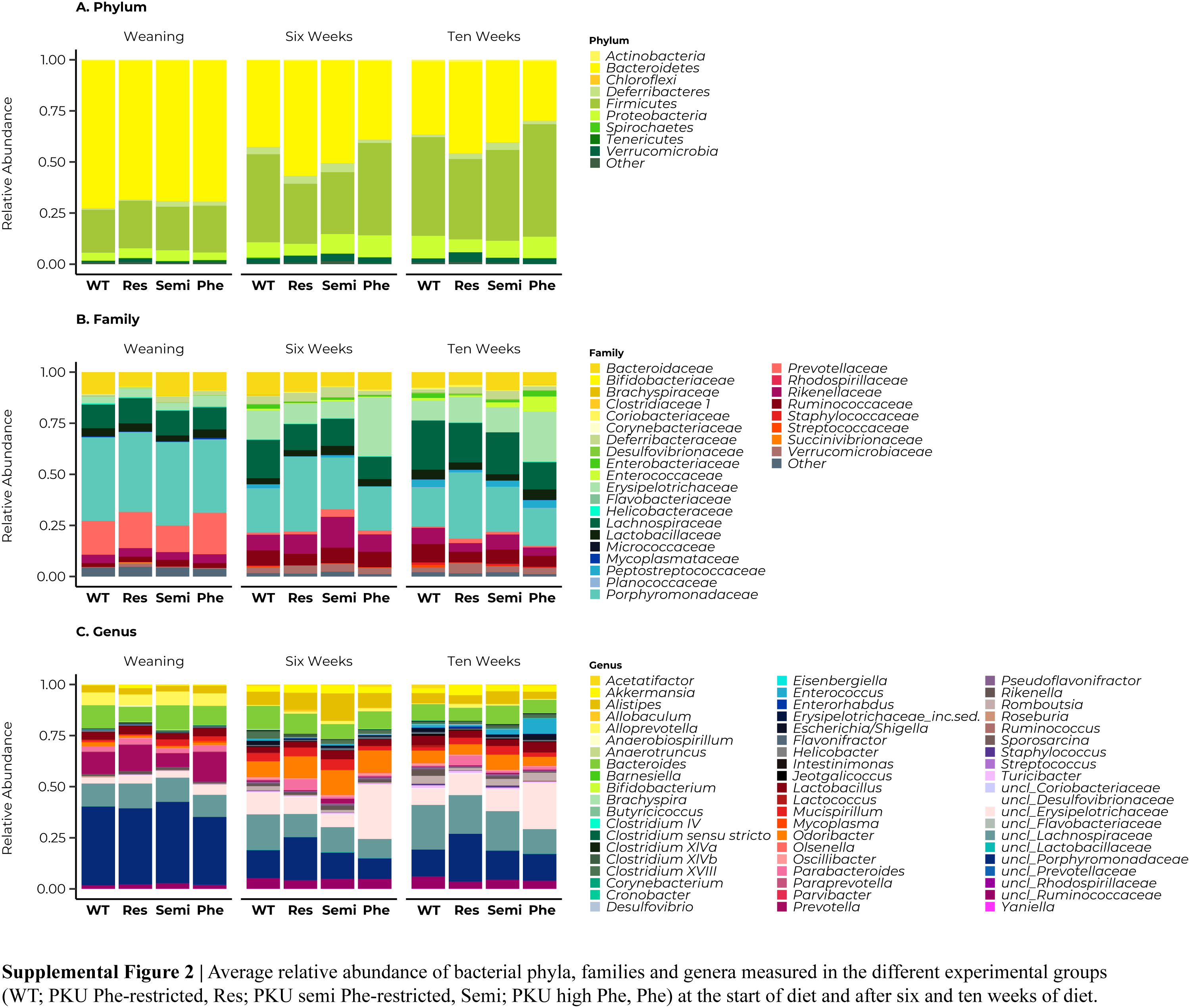

Supplement: Supplementary Figure 2 — Average relative abundance of bacterial phyla, families and genera. [file Image_2.tif]
